# Feasibility and acceptability of persons on long‐acting cabotegravir for HIV prevention in the SEARCH Dynamic Choice HIV Prevention trial extension in rural Kenya and Uganda: a longitudinal cohort study

**DOI:** 10.1002/jia2.26465

**Published:** 2025-07-02

**Authors:** Elijah R. Kakande, Laura B. Balzer, Jane Kabami, James Ayieko, Gabriel Chamie, Nicole Sutter, Helen Sunday, Marilyn Nyabuti, Janice Litunya, Carol Camlin, Jason Johnson‐Peretz, Jenny Temple, Geoff Lavoy, Catherine Koss, Maggie Czarnogorski, Maya L. Petersen, Moses R. Kamya, Diane V. Havlir

**Affiliations:** ^1^ Infectious Diseases Research Collaboration Kampala Uganda; ^2^ Division of Biostatistics University of California Berkeley Berkeley California USA; ^3^ Kenya Medical Research Institute Nairobi Kenya; ^4^ Division of HIV, Infectious Diseases, and Global Medicine University of California San Francisco San Francisco California USA; ^5^ Department of Digital Innovation and Implementation Science ViiV Healthcare Durham North Carolina USA; ^6^ Department of Medicine Makerere University College of Health Sciences Kampala Uganda

**Keywords:** acceptability, dynamic choice, feasibility, HIV prevention, implementation, long‐acting injectable cabotegravir

## Abstract

**Introduction:**

Injectable cabotegravir (CAB‐LA) is highly effective for HIV prevention, but real‐world implementation studies in Africa are ongoing. We assessed feasibility and acceptability among participants who used CAB‐LA in the SEARCH Dynamic Choice HIV Prevention extension study in rural Uganda and Kenya.

**Methods:**

From January 2023 to December 2024, we followed females and males who were aged ≥ 15 years, with self‐assessed risk for HIV acquisition, in the intervention arm of the SEARCH Dynamic Choice HIV Prevention extension study, and received at least one CAB‐LA injection during the first 48 weeks. To assess the feasibility and acceptability of CAB‐LA, we designed quantitative surveys based on the Theoretical Framework for Acceptability. Surveys were administered at CAB‐LA initiation, after 24 and 48 weeks of use, and discontinuation of CAB‐LA.

**Results:**

Of 487 intervention arm participants, 274 (56%) started CAB‐LA (183 females; 91 males; 79 youth aged 15–24 years). Of whom, 264 completed the survey at initiation, 206 after 24 weeks on CAB‐LA, 201 after 48 weeks on CAB‐LA and 69 at discontinuation of CAB‐LA. Most participants (65%; 171/264) reported choosing CAB‐LA because it was easier to take than pills, and nearly all (99%; 261/264) had limited knowledge of CAB‐LA prior to the study. Concerns for side effects were the largest anticipated and experienced barrier to CAB‐LA. Overall and with subgroups, satisfaction with CAB‐LA was high at 24 weeks (97%; 200/206) and 48 weeks (96%; 193/201). Nearly all participants reported that taking CAB‐LA was easy at 24 weeks (95%; 195/206) and 48 weeks (99%; 198/201). At CAB‐LA discontinuation, 83% (57/69) were likely to extremely likely to recommend CAB‐LA to a friend: 80% (20/25) of males, 84% (37/44) of females, 100% (19/19) of youth and 76% (38/50) of older adults.

**Conclusions:**

In rural Uganda and Kenya, over half of participants in the SEARCH trial who were offered choice of oral PrEP/PEP or CAB‐LA chose and started CAB‐LA during the first 48 weeks. For both males and females and younger and older adults, CAB‐LA was both feasible and acceptable to deliver with satisfaction remaining high throughout the study, and nearly all reporting ease of use.

**Clinical Trial Number:**

05549726

## 1 INTRODUCTION

Long‐acting injectable cabotegravir (CAB‐LA) is highly effective for HIV prevention compared to oral pre‐exposure prophylaxis (PrEP); tenofovir disoproxil fumarate/emtricitabine (TDF/FTC) [1, 2] and is an important addition to a rapidly expanding HIV prevention toolkit. However, real‐world implementation data to inform the scaling up of this option in sub‐Saharan Africa (SSA) are needed to understand how to implement this new treatment modality in the real world. There is increasing momentum for HIV prevention delivery using person‐centred, choice‐based approaches [3, 4, 5, 6, 7]. Implementation data are needed to inform decision‐making in countries rolling out CAB‐LA in their HIV prevention programmes with emphasis on innovations tailored to the needs of both males and females, and of both younger and older persons at risk for HIV. Data on the implementation of HIV prevention options among heterosexual males in SSA needed to inform access programmes are particularly scarce, but are underway [8].

In the SEARCH Dynamic Choice HIV Prevention extension study [9], we offered choice of biomedical HIV prevention products including CAB‐LA, oral TDF/FTC and post‐exposure prophylaxis (PEP), with the ability to switch between options over time (Figure S1). This intervention increased biomedical prevention coverage, defined as the proportion of follow‐up time that was covered by a prevention option, by 56.4% compared to the standard of care, which offered oral PrEP/PEP according to national guidelines [9]. Moreover, CAB‐LA increased time at risk covered by a biomedical prevention option, and 42% of CAB‐LA users were not using any biomedical prevention option at the time of initiating CAB‐LA [9]. We evaluated the feasibility and acceptability of CAB‐LA among males and females in the intervention arm of the extension who chose and received CAB‐LA during the first 48 weeks of the extension. Participants in the control arm of the extension study were not considered for participation because CAB‐LA was not available as standard of care in Uganda and Kenya at the time of study implementation. Our analyses aimed to improve our understanding of client perceptions about this long‐acting injectable HIV prevention option.

## 2 METHODS

### 2.1 Study design, site and population

This was an open‐label extension study of the SEARCH Dynamic Choice HIV Prevention trials (Figure S2), comparing a person‐centred intervention that offered two biomedical prevention options (oral PrEP or PEP [for recent exposure or as a pill in pocket for anticipated exposure]) with the option to switch products over time versus the standard‐of‐care among males and females in southwestern Uganda and Western Kenya [6, 7, 9, 10]. The 96‐week extension expanded the intervention to offer three biomedical options: CAB‐LA, oral PrEP and PEP (Figure S1 in Supporting Information) [9]. Eligibility criteria for participation in the extension were prior trial participation, testing HIV negative and residing in the study region. The extension study began enrolment in January 2023, and data collection ended in December 2024. Females who were pregnant at the extension start were not allowed to initiate CAB‐LA. If a woman became pregnant during the study while receiving CAB‐LA, she was provided the option to continue CAB‐LA under informed consent. Our analysis focused on participants in the intervention arm who had chosen and received at least one injection of CAB‐LA within the first 48 weeks of the extension.

### 2.2 Study procedures and follow‐up

We administered structured surveys at CAB‐LA initiation and after 24 and 48 weeks of use to capture client acceptability and feasibility based on anticipated and lived experiences over time. Surveys were developed using some constructs of the Theoretical Framework for Acceptability (TFA) [11], including affective attitude, burden, perceived effectiveness, intervention coherence and self‐efficacy (Table S1 in Supporting Information) to understand anticipated and experienced patient perspectives about CAB‐LA. The survey was designed with the precept that participants had been exposed to the choice of oral PrEP and PEP during the initial trials. At the initiation visit, prior to receiving the first injection, participants selected their reasons for initiating CAB‐LA. Participants could select more than one answer as well as specify “Other” with a free‐text response. Knowledge of CAB‐LA prior to the study was also assessed at the initiation visit. At all visits, we asked about anticipated (at initiation) or experienced (at weeks 24 and 48) barriers to using CAB‐LA; participants could again select more than one answer, specify a free‐text response or report not having barriers. Satisfaction was assessed at weeks 24 and 48. We defined feasibility as ease of using CAB‐LA and assessed it at initiation and after 24 and 48 weeks on CAB‐LA. We assessed acceptability as: (a) reasons for initiating CAB‐LA; (b) knowledge of CAB‐LA; (c) barriers to initiating CAB‐LA; and (d) satisfaction (level of satisfaction with CAB‐LA and likelihood of recommending CAB‐LA to a friend) using constructs of the TFA [12]. We administered an analogous survey to participants when they discontinued CAB‐LA. All surveys were administered by trained healthcare workers who also delivered the SEARCH Dynamic Choice HIV Prevention intervention to study participants.

### 2.3 Statistical measures

We used descriptive statistics to summarize the study population. We summarized responses about reasons for choosing CAB‐LA and anticipated/experienced barriers to using CAB‐LA with numbers and proportions. Free‐text responses to the “Other” category were recategorized, where possible. Implementation outcomes (i.e. knowledge, feasibility and acceptability) were structured using a Likert scale format and summarized using numbers and proportions in each response category. We reported summaries overall and by sex and age group with “younger” as aged 15–24 years and “older” as aged 25+ years.

### 2.4 Ethical approval

The study was reviewed and approved by The Makerere University School of Medicine Research and Ethics Committee, the Uganda National Council for Science and Technology, the Kenya Scientific and Ethics Review Unit, the National Commission for Science, Technology and Innovation, and the University of California San Francisco Committee on Human Research. All participants consented to study participation.

## 3 RESULTS

### 3.1 Baseline characteristics of participants starting CAB‐LA

Of 487 participants in the intervention arm of the extension, 274 (56%) chose and initiated CAB‐LA during the first 48 weeks of the extension study and were the focus of our study on the feasibility and acceptability of CAB‐LA [9]. Among the 274 CAB‐LA users, 250 (91%) initiated CAB‐LA at the start of the extension study. As shown in Table 1, 67% were females, 71% were aged 25 years and above, and 57% lived in Kenya. Eighty‐three percent were married or cohabiting; 41% were farmers; 58% of males reported that they were circumcised; 19% used alcohol; and 18% were highly mobile (defined as being consecutively away for 2 weeks at least twice in the last 12 months).

**Table 1:** Baseline characteristics of study participants who started CAB‐LA, overall and by sex and age group

	Females	Males	15−24 years	25+ years	Overall
	*n* = 183	*n* = 91	*n* = 79	*n* = 195	*N* = 274
**Sex**					
Females	183 (100%)		54 (68%)	129 (66%)	183 (67%)
Males		91 (100%)	25 (32%)	66 (34%)	91 (33%)
**Age**					
Aged 15–24 years	54 (30%)	25 (27%)	79 (100%)		79 (29%)
Aged 25+ years	129 (70%)	66 (73%)		195 (100%)	195 (71%)
**Country**					
Kenyan	106 (58%)	51 (56%)	55 (70%)	102 (52%)	157 (57%)
Ugandan	77 (42%)	40 (44%)	24 (30%)	93 (48%)	117 (43%)
**Recruitment setting**					
Antenatal clinic	81 (44%)	0 (0%)	34 (43%)	47 (24%)	81 (30%)
Outpatient department	43 (23%)	42 (46%)	22 (28%)	63 (32%)	85 (31%)
Community	59 (32%)	49 (54%)	23 (29%)	85 (44%)	108 (39%)
**Marital status**					
Single (unmarried)	12 (7%)	19 (21%)	25 (32%)	6 (3%)	31 (11%)
Married/cohabitating	157 (86%)	71 (78%)	53 (67%)	175 (90%)	228 (83%)
Divorced/separated/widowed	14 (8%)	1 (1%)	1 (1%)	14 (7%)	15 (5%)
**Occupation**					
Farmer	81 (44%)	31 (34%)	19 (24%)	93 (48%)	112 (41%)
Shopkeeper/market vendor	24 (13%)	4 (4%)	6 (8%)	22 (11%)	28 (10%)
Student	8 (4%)	4 (4%)	11 (14%)	1 (1%)	12 (4%)
Manual labour/construction	1 (1%)	14 (15%)	5 (6%)	10 (5%)	15 (5%)
Transportation	0 (0%)	10 (11%)	6 (8%)	4 (2%)	10 (4%)
Bar/hotel/restaurant	2 (1%)	3 (3%)	1 (1%)	4 (2%)	5 (2%)
Fishing/fishmonger	3 (2%)	2 (2%)	0 (0%)	5 (3%)	5 (2%)
**Highest education**					
Less than primary	59 (32%)	28 (31%)	19 (24%)	68 (35%)	87 (32%)
Primary	87 (48%)	34 (37%)	34 (43%)	87 (45%)	121 (44%)
Secondary	27 (15%)	15 (16%)	21 (27%)	21 (11%)	42 (15%)
Post‐secondary	10 (5%)	14 (15%)	5 (6%)	19 (10%)	24 (9%)
**Alcohol use** ^a^	21 (11%)	32 (35%)	10 (13%)	43 (22%)	53 (19%)
**Highly mobile**	21 (11%)	28 (31%)	16 (20%)	33 (17%)	49 (18%)
**Pregnant** (females only)	7 (4%)		2 (4%)	5 (4%)	7 (4%)
**Circumcised** (males only)		53 (58%)	18 (72%)	35 (53%)	53 (58%)

^a^Alcohol use was defined as self‐reported use of any alcohol. Persons were highly mobile if they were consecutively away for 2 weeks at least twice in the last 12 months.

**Figure 1:**
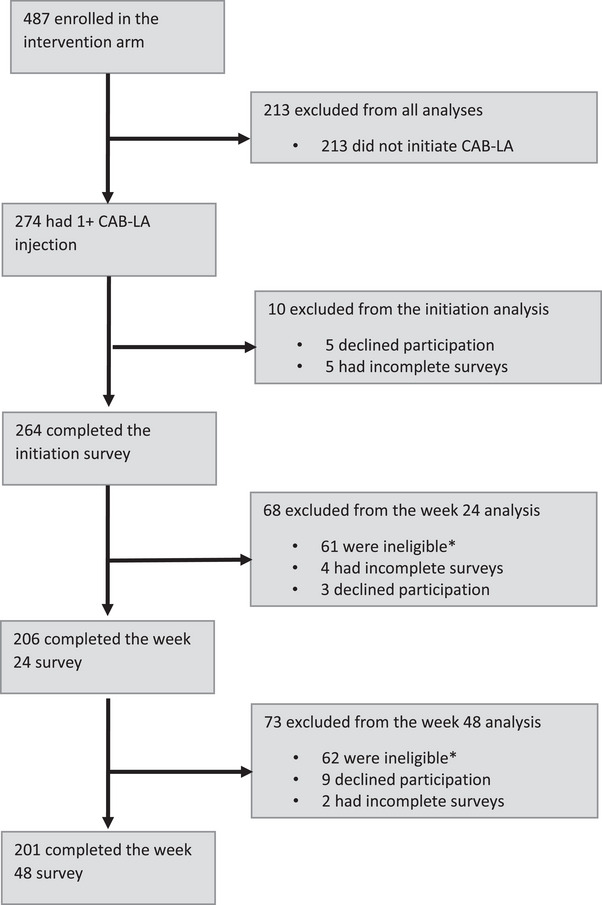
**CONSORT diagram**. ^*^Ineligible if they had discontinued CAB‐LA or were bridging with another product. Abbreviation: CAB‐LA, long‐acting injectable cabotegravir.

As shown in Figure 1, 264/274 (96%) of participants completed the survey at CAB‐LA initiation. By week 24, 61/274 (22%) of participants had discontinued CAB‐LA and were not eligible for the survey; 206/213 (97%) of eligible participants completed the week 24 survey. By week 48, 62/274 (23%) of participants had discontinued CAB‐LA and were ineligible for the survey, 201/212 (95%) of eligible participants completed the week 48 survey.

### 3.2 Acceptability of CAB‐LA

#### 3.2.1 Reasons for initiating CAB‐LA

Overall, 65% chose CAB‐LA because it was easier to get an injection than to take pills, similar in both sexes (66% of females and 62% of males) and age groups (58% of youth and 68% of older adults; Table 2). Almost half of the participants (49%) preferred CAB‐LA because they will forget to take pills. Thirty‐one percent of participants chose CAB‐LA because of stigma, and this reason was similar among females (33%) and males (27%), but more pronounced among youth (42%) than older persons (26%). Other reasons for choosing CAB‐LA included the side effects of pills (18%) and discretion afforded by CAB‐LA (16%).

**Table 2:** Reasons for initiating CAB‐LA, overall and by sex and age group^a^

	Overall	Females	Males	15−24 years	25+years
Response	(*N* = 264)	(*n* = 175)	(*n* = 89)	(*n* = 76)	(*n* = 188)
Easier to get an injection than to take pills	171 (65%)	116 (66%)	55 (62%)	44 (58%)	127 (68%)
Do not want to take pills because I cannot remember	130 (49%)	80 (46%)	50 (56%)	38 (50%)	92 (49%)
Do not want to take pills because someone will know I am taking them	81 (31%)	57 (33%)	24 (27%)	32 (42%)	49 (26%)
Side effects of pills	48 (18%)	34 (19%)	14 (16%)	13 (17%)	35 (19%)
Cannot take pills because my partner or friends will not let me	41 (16%)	30 (17%)	11 (12%)	17 (22%)	24 (13%)
Other	15 (6%)	9 (5%)	6 (7%)	3 (4%)	12 (6%)

^a^ Participants could choose more than one response.

#### 3.2.2 Knowledge of CAB‐LA

Prior to learning about it in the study, 99% (261/264) of participants had basic to no knowledge of CAB‐LA, consistent by sex (99% of females and males) and across age groups (100% of youth and 98% of older persons).

#### 3.2.3 Barriers to using CAB‐LA

At CAB‐LA initiation, 13% (35/264) did not anticipate any barriers to initiating CAB‐LA (Table 3). Among those who anticipated barriers (229/264), 66% (152/229) were concerned about side effects. Concerns for side effects were slightly more pronounced among females than males (69% vs. 60%) and for younger than older adults (76% vs. 62%). In addition, missing appointments due to travel, 17% (39/229), and forgetting appointments, 13% (30/229), were also reported as anticipated barriers to CAB‐LA use. Only 4% (9/229) mentioned stigma with CAB‐LA injections as a concern, with similar proportions for both females and males (5% and 3%, respectively) and younger and older persons (1% and 5%, respectively).

**Table 3:** Anticipated barriers to starting CAB‐LA, overall and by sex and age group, asked the CAB‐LA initiation visit^a^

	Overall	Females	Males	15−24 years	25+ years
Response	(*N* = 264)	(*n* = 175)	(*n* = 89)	(*n* = 76)	(*n* = 188)
No barriers reported	35 (13%)	21 (12%)	14 (16%)	9 (12%)	26 (14%)
Among those reporting barriers^a^	*N* = 229	*N* = 154	*N* = 75	*N* = 67	*N* = 101
Side effects of the injections	152 (66%)	107 (69%)	45 (60%)	51 (76%)	101 (62%)
Missing my appointments because I am travelling	39 (17%)	21 (14%)	18 (24%)	12 (18%)	27 (17%)
Missing my appointments because I forget	30 (13%)	21 (14%)	9 (12%)	6 (9%)	24 (15%)
Missing appointments because of transportation challenges	14 (6%)	5 (3%)	9 (12%)	3 (4%)	11 (7%)
Persons (partner or friends) knowing I am taking an injection and telling others	9 (4%)	7 (5%)	2 (3%)	1 (1%)	8 (5%)
Missing appointments because of their frequency	4 (2%)	3 (2%)	1 (1%)	1 (1%)	3 (2%)
Failure to take the injection because of illness	5 (2%)	4 (3%)	1 (1%)	0 (0%)	5 (3%)
Other	1 (0%)	0 (0%)	1 (1%)	0 (0%)	1 (1%)
Restrictive or unsupportive partner	1 (0%)	1 (1%)	0 (0%)	0 (0%)	1 (1%)

^a^ Participants could choose more than one response.

A notable proportion of participants reported experiencing no barriers to CAB‐LA: 21% (43/206) after 24 weeks on CAB‐LA and 19% (38/201) after 48 weeks (Tables S2 and S3 of the Supporting Information). Side effects were the most reported barrier once people had experience with CAB‐LA and were reported by 70% (114/163) after 24 weeks on CAB‐LA and 65% (106/163) after 48 weeks.

#### 3.2.4 Satisfaction

##### 3.2.4.1 Level of satisfaction with using CAB‐LA

**Figure 2:**
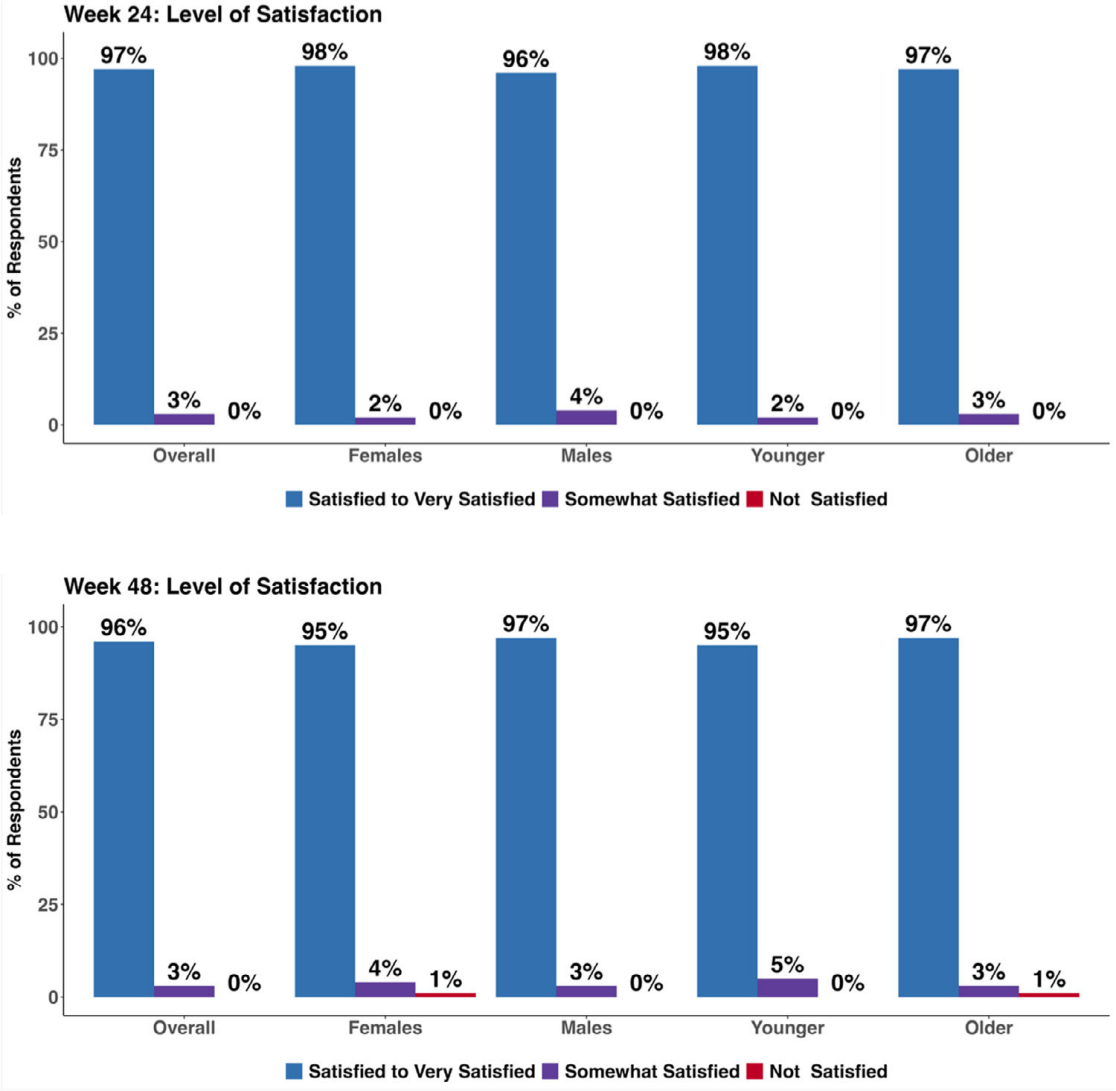
**Level of satisfaction with long‐acting injectable cabotegravir (CAB‐LA) over time, by sex and by age group**.

After 24 weeks of use, 97% (201/207) of CAB‐LA users were Satisfied to Very Satisfied with CAB‐LA as an HIV biomedical prevention option. Levels of satisfaction remained high at 96% (188/196) after 48 weeks on CAB‐LA. Similarly, high levels of satisfaction were observed by sex and age group (Figure 2). At 24 weeks, 98% of females, 96% of males, 98% of younger adults and 97% of older adults were Satisfied to Very Satisfied with CAB‐LA. At 48 weeks, these proportions were 95%, 97%, 95% and 96%, respectively.

##### 3.2.4.2 Likelihood of recommending CAB‐LA to a friend

**Figure 3:**
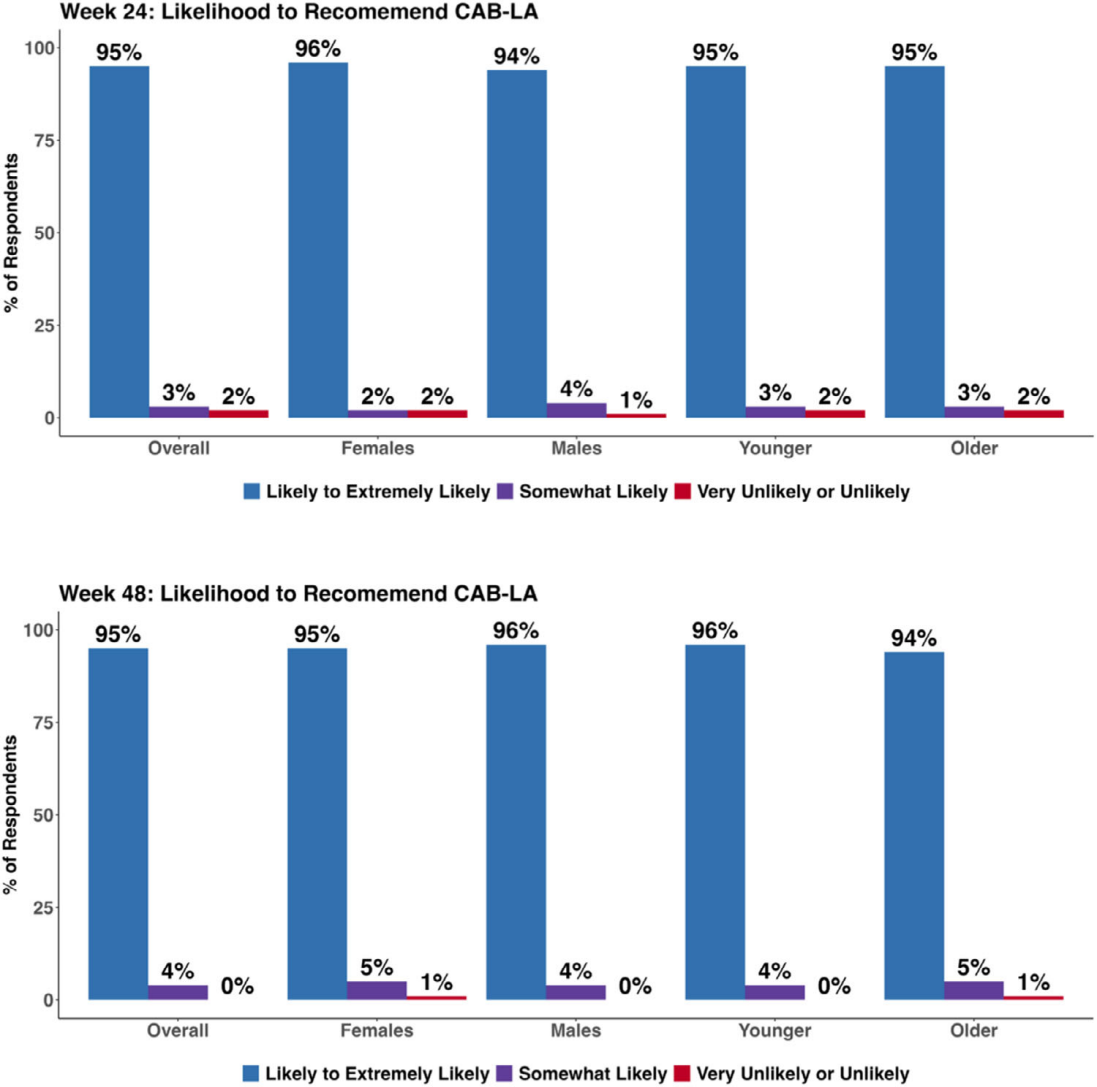
**Likelihood of recommending long‐acting injectable cabotegravir (CAB‐LA) over time, by sex and age group**.

After 24 weeks on CAB‐LA, 95% (197/207) of participants were Likely to Highly Likely to recommend CAB‐LA to a friend, persisting at 95% (186/196) through 48 weeks after initiation. Similar findings over time were observed among females (96% and 94%), males (94% and 96%), youth (95% and 96%) and older participants (95% and 94%; Figure 3).

### 3.3 Feasibility of using CAB‐LA

**Figure 4:**
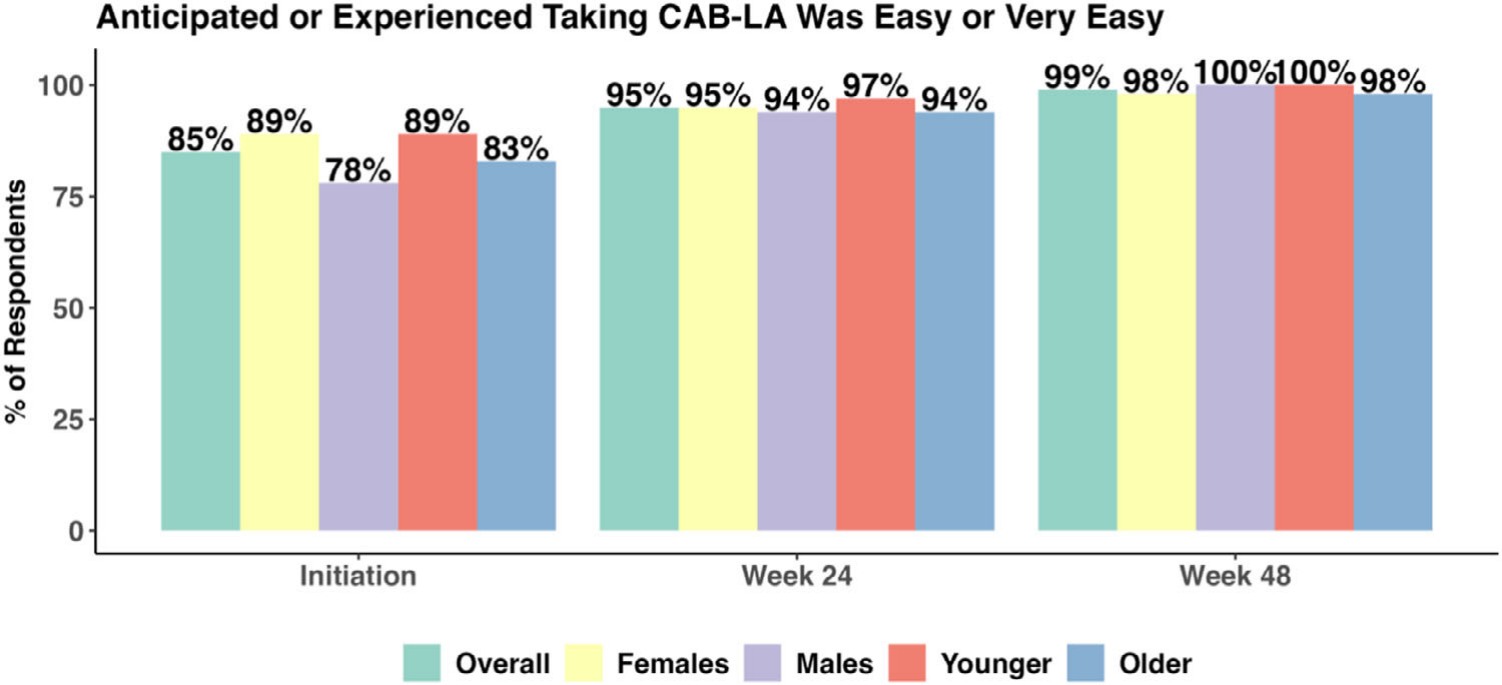
**Over time, the proportion of participants reporting that they anticipated (initiation) or experienced (at 24 or 48 weeks) that it would be or was Easy or Very Easy to take CAB‐LA, overall and by sex and age group**. Abbreviation: CAB‐LA, long‐acting injectable cabotegravir.

At initiation but prior to receiving their first injection, 85% (224/264) anticipated that it would be Easy to Very Easy to use CAB‐LA (Figure 4). Most females (89%), males (78%), youth (89%) and older participants (83%) anticipated that it would be Easy to Very Easy to take CAB‐LA. Overall, the proportion of participants who found it Easy to Very Easy to use CAB‐LA increased to 95% (195/206) after 24 weeks and to 99% (198/201) after 48 weeks following the first injection. Similar increases were observed over time among females (95% and 98%), males (94% and 100%), youth (97% and 1000%) and older persons (94% and 98%).

### 3.4 Survey results among participants who discontinued CAB‐LA

Overall, 69/274 (25%) participants discontinued CAB‐LA and completed a survey at discontinuation. Twelve percent (8/69) reported no barriers at discontinuation. Among those who reported barriers, the most common barrier was side effects: 70% (43/61). Other barriers included missing appointments because of travel (36%), missing appointments because of their frequency (33%), forgetting appointments (10%), concerns that friends will know that they are taking an injection and tell others (8%), and transportation challenges (3%). Seventy‐four percent (51/69) were satisfied to very satisfied with CAB‐LA; 83% (57/69) would recommend CAB‐LA to a friend, and 81% (56/69) found it Easy to Very Easy to use CAB‐LA (Table 4).

**Table 4:** Satisfaction, ease of use and likelihood to recommend long‐acting injectable cabotegravir among participants who discontinued it

	Overall	Females	Males	15−24 years	25+ years
Response	(*N* = 69)	(*n* = 44)	(*n* = 25)	(*n* = 19)	(*n* = 50)
**Satisfaction with CAB‐LA**					
Satisfied to Very Satisfied	51 (74%)	33 (75%)	18 (72%)	14 (74%)	37 (74%)
Somewhat Satisfied	9 (13%)	6 (14%)	3 (12%)	3 (16%)	6 (12%)
Not Satisfied	9 (13%)	5 (11%)	4 (16%)	2 (11%)	7 (14%)
**Ease of use**					
Easy to Very Easy	56 (81%)	37 (84%)	19 (76%)	17 (89%)	39 (78%)
Somewhat Easy	9 (13%)	6 (14%)	3 (12%)	2 (11%)	7 (14%)
Very Difficult to Difficult	4 (6%)	1 (2%)	3 (12%)	0 (0%)	4 (8%)
**Likelihood to recommend CAB‐LA**					
Likely to Extremely Likely	57 (83%)	37 (84%)	20 (80%)	19 (100%)	38 (76%)
Somewhat Likely	10 (14%)	5 (11%)	5 (20%)	0 (0%)	10 (20%)
Very Unlikely or Unlikely	2 (3%)	2 (5%)	0 (0%)	0 (0%)	2 (4%)
Likely to Extremely Likely	57 (83%)	37 (84%)	20 (80%)	19 (100%)	38 (76%)

Abbreviation: CAB‐LA, long‐acting injectable cabotegravir.

## 4 DISCUSSION

In this implementation study, males and females who used CAB‐LA in the SEARCH Dynamic Choice HIV Prevention extension study shared their perceptions of the knowledge, feasibility and acceptability of CAB‐LA based on anticipated (at initiation) and lived experiences during study follow‐up. CAB‐LA offered in a person‐centred Dynamic Choice HIV Prevention model was highly feasible and acceptable for both males and females and for youth and older persons with high levels of satisfaction sustained throughout.

Most CAB‐LA users in the study preferred it to oral options because of the ease of taking injections compared to pills and because they were likely to forget to take their daily oral pill compared to taking an injection once every 2 months. In hypothetical studies [13, 14], injectable PrEP was viewed as a solution to poor adherence [15, 16], one of the major drawbacks of oral PrEP, affecting its effectiveness in head‐to‐head efficacy trials [1, 2, 17]. The discretion offered by CAB‐LA compared to oral pills has been highlighted in multiple studies [18, 19, 20]. In our previous qualitative work [21], fear of partners and neighbours discovering that females were taking oral PrEP and the influence of power dynamics were prominent themes that could explain why more females than males reported stigma as the reason for preferring CAB‐LA. The perceptions of youth towards CAB‐LA are less well described, although implementation studies are currently ongoing. In oral PrEP studies, PrEP uptake and adherence in youth was hampered by stigma [22, 23]. Our study suggests that CAB‐LA responds to the challenge of stigma in this group. In our qualitative work, participants in the dynamic choice HIV prevention studies appreciated oral PrEP as a biomedical option, albeit amid challenges of stigma, particularly the fact that oral PrEP may be mistaken for anti‐retroviral therapy (ART), and the unwillingness of partners to support decisions to use oral PrEP [21]. CAB‐LA was viewed as an alternative that is distinct from ART and, therefore, not subject to the same stigma [24].

Prior to this study, almost all participants did not know about CAB‐LA as a biomedical HIV prevention option, consistent across age groups and sex. At the time of study implementation, CAB‐LA had not yet been approved for HIV prevention in Uganda or Kenya, which may have contributed to the very low knowledge observed in the study. Our findings are similar to what others have reported in SSA [25], with CAB‐LA approved in only a handful of countries in SSA and an even smaller number of countries rolling out CAB‐LA within their HIV prevention programmes. Since knowledge and multiple options are important precursors in ensuring that clients have true freedom of choice [26], it is important that information about new biomedical HIV prevention options such as CAB‐LA is widely disseminated to generate demand for CAB‐LA.

Almost half of study participants anticipated that side effects would be a barrier to starting CAB‐LA and side effects remained the most commonly experienced barrier. Some participants anticipated and experienced structural barriers such as forgetting appointments and travel, similar to what others have reported [27]. As part of the Dynamic Choice HIV Prevention intervention, participants received telephone reminders to address the barrier of forgetting appointments [28]. For highly mobile persons, 2 monthly appointments may be challenging to keep. How best to implement CAB‐LA in highly mobile populations remains an open implementation question. Importantly, a notable proportion of participants did not experience any barriers at all with CAB‐LA.

At initiation, the vast majority of CAB‐LA users anticipated that it would be easy to use CAB‐LA, consistent for both females and males, and for youth and adults 25 years and above. Data on the implementation of CAB‐LA for HIV prevention in SSA are still being generated; however, in studies evaluating people's preferences for HIV prevention options, some participants were open to the idea of using CAB‐LA for HIV prevention [19, 29, 30], similar to our findings. After 24 weeks of use, the majority of participants reported that it had been easy to use CAB‐LA. This persisted after 48 weeks of use and was consistent across age groups and sex, suggesting that it is highly feasible to implement CAB‐LA. Our person‐centred SEARCH Dynamic Choice HIV Prevention intervention was designed to address barriers to the uptake of HIV biomedical prevention options through the development of personalized plans and enhanced access to providers [9]. It is plausible that the intervention contributed to the ease of use of CAB‐LA.

Participants receiving CAB‐LA in this study, including females and males, youth and adults 25 years or more, were highly satisfied with the product and willing to recommend it to a friend. These observations persisted from week 24 to week 48 of follow‐up. To our knowledge, this is the first study to report on the experiences of heterosexual men using CAB‐LA in SSA. Most CAB‐LA implementation studies have been conducted among men who have sex with men in America and Europe [30]. In our study, CAB‐LA led to high levels of satisfaction among males in SSA.

Participants completed a survey about their experiences when they discontinued CAB‐LA. Among this group, side effects were the most common barrier experienced with CAB‐LA, as expected. Importantly, acceptability of CAB‐LA remained high in this group, with the majority satisfied with CAB‐LA and willing to recommend it to a friend, suggesting that although these participants reported experiencing barriers with CAB‐LA, their overall experience with this biomedical option was good. This may also explain why acceptability was so high among participants who remained on CAB‐LA in the study.

Our study has some limitations. First, we did not administer surveys to persons who did not initiate CAB‐LA. Instead, our focus was on the anticipated and lived experiences of males and females who had initiated CAB‐LA. Our study design allowed participants to discontinue and restart CAB‐LA at any time during follow‐up and among participants who discontinued CAB‐LA, feasibility and acceptability remained high. Second, our data are limited to persons who discontinued CAB‐LA during the follow‐up. However, among the 69 participants who completed a survey at discontinuation, the acceptability of CAB‐LA was high. Nevertheless, we cannot rule out that our surveys missed persons for whom CAB‐LA was a less feasible or acceptable option. Third, we did not include data on providers to understand their perspectives on CAB‐LA implementation. Understanding feasibility and acceptability from a healthcare provider's standpoint is important to inform the implementation of CAB‐LA in this setting. Finally, longer follow‐up could provide evolving or new challenges.

## 5 CONCLUSIONS

In rural Uganda and Kenya, over half of the participants who were offered the choice of oral PrEP/PEP or CAB‐LA chose and started CAB‐LA during the first 48 weeks in the SEARCH Dynamic Choice HIV Prevention extension study. From clients’ anticipated and lived experiences, CAB‐LA was a feasible and acceptable HIV prevention choice with satisfaction remaining high throughout the study and a high ease of use reported.

## COMPETING INTERESTS

DVH reports non‐financial support from ViiV Healthcare. All other authors declare no conflicts of interest.

## AUTHORS’ CONTRIBUTIONS

ERK, LBB, JK, JA, GC, NS, HS, JJ‐P, MN, JL, MRK, MLP and DVH conceptualized and designed the study. All authors participated in study operations or data collection. JT, GL and LBB performed the data analyses with input from ERK, JA, HS, MC, MN, JL, GC, JK, CK, CC, NS, GC, MRK, DVH and MLP. ERK and DVH developed the initial draft of the manuscript. MLP, MRK and LBB developed the manuscript further with input from the other authors. All authors reviewed and approved the final manuscript.

## FUNDING

Research reported in this manuscript was supported by the U.S. National Institute of Allergy and Infectious Diseases (NIAID), the National Heart, Lung, and Blood Institute (NHLBI), and the National Institute of Mental Health (NIMH) and co‐funded under award number U01AI150510.

## DISCLAIMER

The content is solely the responsibility of the authors and does not necessarily represent the official views of the NIH.

## Supporting information




**Table S1**: CAB‐LA survey questions mapped onto constructs of the Theoretical Framework of Acceptability

**Table S2**: Barriers faced at week 24

**Table S3**: Barriers faced at week‐48

**Figure S1**: Dynamic Choice Prevention intervention

**Figure S2**: Illustration of overall study design

## Data Availability

A complete de‐identified patient dataset sufficient to reproduce the study findings will be made available approximately 1 year after completion of the ongoing trial (NCT05549726), following approval of a concept sheet summarizing the analyses to be performed. Further inquiries can be directed to the SEARCH Scientific Committee at douglas.black@ucsf.edu.
