# Supplementary material for: Feasibility and acceptability of persons on long‐acting cabotegravir for HIV prevention in the SEARCH Dynamic Choice HIV Prevention trial extension in rural Kenya and Uganda: a longitudinal cohort study
Source: J Int AIDS Soc. 2025 Jul 2;28(Suppl 2):e26465. doi: 10.1002/jia2.26465 (PMC12215826; doi:10.1002/jia2.26465)
Supplement: Supplementary file 1 — Table S1: CAB‐LA survey questions mapped onto constructs of the Theoretical Framework of Acceptability [file JIA2-28-e26465-s004.docx]

**Supplementary Table S1: CAB-LA survey questions mapped onto constructs of the Theoretical Framework of Acceptability.**

| **Construct** | **Questions and responses included in the survey** |
| --- | --- |
| **Affective attitude:** How an individual feels about the intervention | - What is the likelihood of you recommending CAB-LA to a friend? (Very Unlikely; Unlikely; Somewhat Likely; Likely; Extremely Likely) |
| **Burden:** Perceived amount of effort that is required to participate in the intervention | - How easy was it to take CAB-LA? (Very Difficult; Difficult; Somewhat Easy; Easy; Very Easy) |
| **Perceived effectiveness:** The extent to which the intervention is perceived as likely to achieve its purpose | - What is your level of satisfaction for using CAB-LA? (Not Very Satisfied; Not Satisfied; Somewhat Satisfied; Satisfied; Very Satisfied) |
| **Ethicality:** The extent to which the intervention has good fit with an individual’s value system |  |
| **Intervention coherence:** The extent to which the participant understands the intervention and how it works | - How much did you know about CAB-LA before you were told about this option in the study?   (No Information; Minimal Information; Basic Information; Adequate Information; A lot of Information) |
| **Opportunity costs:** The extent to which benefits, profits or values must be given up to engage in the intervention. | - What concerns or barriers do you anticipate starting CAB LA?  *Select all that apply including “Other”.* - What concerns or barriers did you face with CAB-LA? *Select all that apply including “Other”.* |
| **Self-efficacy:** The participant’s confidence that they can perform the behavior required to participate in the intervention | - What is the main reason you switched to CAB-LA? Are there any other reasons you switched to CAB-LA? *Select all that apply including “Other”.* |
