# Supplementary material for: Feasibility and acceptability of persons on long‐acting cabotegravir for HIV prevention in the SEARCH Dynamic Choice HIV Prevention trial extension in rural Kenya and Uganda: a longitudinal cohort study
Source: J Int AIDS Soc. 2025 Jul 2;28(Suppl 2):e26465. doi: 10.1002/jia2.26465 (PMC12215826; doi:10.1002/jia2.26465)
Supplement: Supplementary file 2 — Table S2: Barriers faced at week 24 [file JIA2-28-e26465-s001.docx]

**Supplementary Table S2: Barriers faced at week-24**

| **Response** | **Overall** | **Females** | **Males** | **Age 15-24 years** | **Age 25+ years** |
| --- | --- | --- | --- | --- | --- |
| No barriers reported | 21% (43/206) | 24% (32/136) | 16% (11/70) | 16% (9/58) | 23% (34/148) |
| Among those reporting barriers |  |  |  |  |  |
| Side effects of the injections | 70% (114/163) | 68% (71/104) | 73% (43/59) | 73% (36/49) | 68% (78/114) |
| Missing appointments because of their frequency | 20% (33/163) | 20% (21/104) | 20% (12/59) | 20% (10/49) | 20% (23/114) |
| Missing my appointments because I am travelling | 17% (28/163) | 12% (13/104) | 25% (15/59) | 10% (5/49) | 20% (23/114) |
| Missing my appointments because I forget | 12% (19/163) | 15% (16/104) | 5% (3/59) | 8% (4/49) | 13% (15/114) |
| Missing appointments because of transportation challenges | 11% (18/163) | 11% (11/104) | 12% (7/59) | 8% (4/49) | 12% (14/114) |
| Persons (partner or friends) knowing I am taking an injection and telling others | 4% (7/163) | 6% (6/104) | 2% (1/59) | 2% (1/49) | 5% (6/114) |
| Other | 4% (6/163) | 5% (5/104) | 2% (1/59) | 2% (1/49) | 4% (5/114) |
| Restrictive or unsupportive partner | 1% (2/163) | 2% (2/104) | 0% (0/59) | 2% (1/49) | 1% (1/114) |
| Failure to take the injection because of illness | 0% (0/163) | 0% (0/104) | 0% (0/59) | 0% (0/49) | 0% (0/114) |
