# Supplementary material for: Feasibility and acceptability of persons on long‐acting cabotegravir for HIV prevention in the SEARCH Dynamic Choice HIV Prevention trial extension in rural Kenya and Uganda: a longitudinal cohort study
Source: J Int AIDS Soc. 2025 Jul 2;28(Suppl 2):e26465. doi: 10.1002/jia2.26465 (PMC12215826; doi:10.1002/jia2.26465)
Supplement: Supplementary file 3 — Table S3: Barriers faced at week‐48 [file JIA2-28-e26465-s002.docx]

| **Response** | **Overall** | **Females** | **Males** | **Age 15-24 years** | **Age 25+ years** |
| --- | --- | --- | --- | --- | --- |
| No barriers reported | 19% (38/201) | 19% (25/130) | 18% (13/71) | 19% (11/57) | 19% (27/144) |
| Among those reporting barriers |  |  |  |  |  |
| Side effects of the injections | 65% (106/163) | 63% (66/105) | 69% (40/58) | 67% (31/46) | 64% (75/117) |
| Missing appointments because of their frequency | 26% (42/163) | 26% (27/105) | 26% (15/58) | 20% (9/46) | 28% (33/117) |
| Missing my appointments because I am travelling | 18% (29/163) | 19% (20/105) | 16% (9/58) | 20% (9/46) | 17% (20/117) |
| Missing my appointments because I forget | 14% (23/163) | 14% (15/105) | 14% (8/58) | 7% (3/46) | 17% (20/117) |
| Missing appointments because of transportation challenges | 11% (18/163) | 9% (9/105) | 16% (9/58) | 7% (3/46) | 13% (15/117) |
| Persons (partner or friends) knowing I am taking an injection and telling others | 6% (10/163) | 10% (10/105) | 0% (0/58) | 2% (1/46) | 8% (9/117) |
| Restrictive or unsupportive partner | 2% (3/163) | 3% (3/105) | 0% (0/58) | 2% (1/46) | 2% (2/117) |
| Other | 0% (0/163) | 0% (0/105) | 0% (0/58) | 0% (0/46) | 0% (0/117) |
| Failure to take the injection because of illness | 0% (0/163) | 0% (0/105) | 0% (0/58) | 0% (0/46) | 0% (0/117) |

**Supplementary Table S3: Barriers faced at week-48**
