# Supplementary material for: Feasibility and acceptability of persons on long‐acting cabotegravir for HIV prevention in the SEARCH Dynamic Choice HIV Prevention trial extension in rural Kenya and Uganda: a longitudinal cohort study
Source: J Int AIDS Soc. 2025 Jul 2;28(Suppl 2):e26465. doi: 10.1002/jia2.26465 (PMC12215826; doi:10.1002/jia2.26465)
Supplement: Supplementary file 4 — Figure S1: Dynamic Choice Prevention intervention [file JIA2-28-e26465-s003.pdf]

Intervention: Dynamic Choice HIV Prevention

PRODUCT CHOICE[1]
(+ option to switch products)
- Oral PrEP (TDF/XTC)
- PEP (pill in pocket)
- *CAB-LA

SERVICE LOCATION CHOICE
- Clinic
- Home / Community site
- Phone/virtual visit

HIV TESTING CHOICE[2]
- Rapid test
- HIV self-test option

REFILL CHOICE
- Up to 3-month PrEP refill**

PATIENT-CENTRED CARE

- **Structured assessment of barriers** to PrEP/PEP start/adherence, with **personalized plans** developed in response
- **Phone access to clinician** for PEP or PrEP starts, advice/questions (24hrs/7 days/week, including holidays)
- **STI service integration at ANC & OPD** (or referral by VHT/CHV)
- **Psychological support** – referrals to counseling for trauma/gender-based violence

PROVIDER TRAINING IN PATIENT CENTERED CARE, PATIENT EDUCATION

1. All clients offered condoms, lubricant.
If no product started at baseline, clients were followed for HIV testing and option to start PrEP/PEP/CAB-LA/ring anytime during follow-up.
2. Rapid blood-based HIV testing first-line. Option for HIV self-testing during follow-up (including PrEP/PEP monitoring).
* CAB-LA injections are restricted to the clinic
** If supplies available, with preference for longer fill if planning not to have in-person visit at week 4

Control: Standard-of-care
- Referral to HIV clinic for standard PrEP & PEP services
