# Supplementary material for: Feasibility and acceptability of persons on long‐acting cabotegravir for HIV prevention in the SEARCH Dynamic Choice HIV Prevention trial extension in rural Kenya and Uganda: a longitudinal cohort study
Source: J Int AIDS Soc. 2025 Jul 2;28(Suppl 2):e26465. doi: 10.1002/jia2.26465 (PMC12215826; doi:10.1002/jia2.26465)
Supplement: Supplementary file 5 — Figure S2: Illustration of overall study design [file JIA2-28-e26465-s005.pptx]

## Slide 1
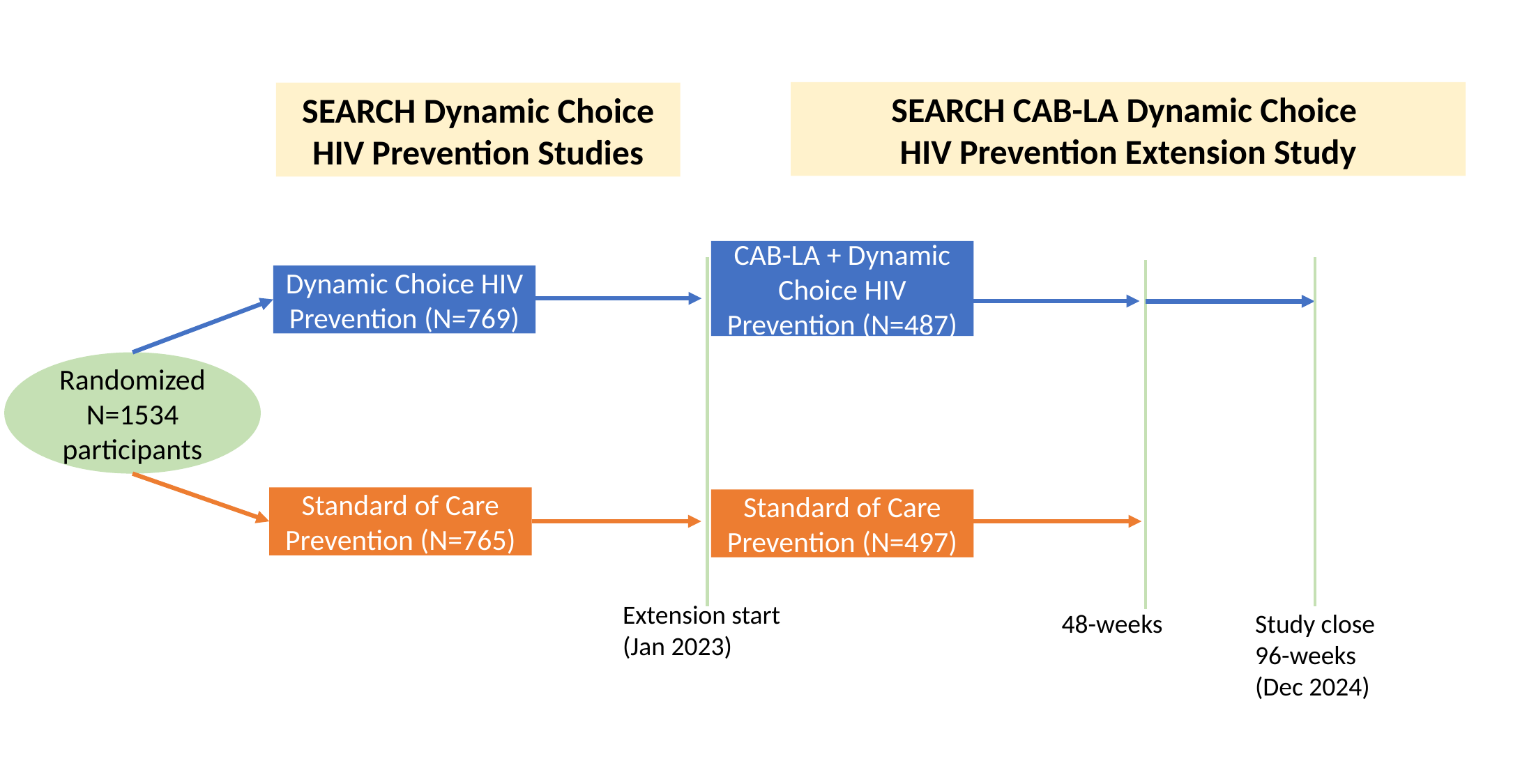

SEARCH CAB-LA Dynamic Choice
HIV Prevention Extension Study
SEARCH Dynamic Choice HIV Prevention Studies
CAB-LA + Dynamic Choice HIV Prevention (N=487)
Dynamic Choice HIV Prevention (N=769)
Randomized
N=1534 participants
Standard of Care Prevention (N=765)
Standard of Care Prevention (N=497)
Extension start
(Jan 2023)
Study close
96-weeks
(Dec 2024)
48-weeks
